# The standards of reporting randomized trials in pets (PetSORT): Methods and development processes

**DOI:** 10.3389/fvets.2023.1137774

**Published:** 2023-03-30

**Authors:** Audrey Ruple, Jan M. Sargeant, Laura E. Selmic, Annette M. O'Connor

**Affiliations:** ^1^Department of Population Health Sciences, Virginia-Maryland College of Veterinary Medicine, Virginia Tech, Blacksburg, VA, United States; ^2^Department of Population Medicine, Ontario Veterinary College, University of Guelph, Guelph, ON, Canada; ^3^Department of Veterinary Clinical Sciences, College of Veterinary Medicine, The Ohio State University, Columbus, OH, United States; ^4^Department of Large Animal Clinical Sciences, College of Veterinary Medicine, Michigan State University, East Lansing, MI, United States

**Keywords:** animal reporting guideline, animal health, randomized trials, small animal clinical trials, companion animals

## Abstract

**Background:**

Reporting of clinical trials conducted in client- and shelter-owned dog and cat populations is not optimal, which inhibits the ability to assess the reliability and validity of trial findings and precludes the ability to include some trials in evidence synthesis.

**Objective:**

To develop a reporting guideline for parallel group and crossover trials that addresses the unique features and reporting requirements for trials conducted in client- and shelter-owned dog and cat populations.

**Design:**

Consensus statement.

**Setting:**

Virtual.

**Participants:**

Fifty-six experts from North America, the United Kingdom, Europe, and Australia working in academia, government (research and regulatory agencies), industry, and clinical veterinary practice.

**Methods:**

A steering committee created a draft checklist for reporting criteria based upon the CONSORT statement and the CONSORT extensions for reporting of abstracts and crossover trials. Each item was presented to the expert participants and was modified and presented again until >85% of participants were in agreement about the inclusion and wording of each item in the checklist.

**Results:**

The final PetSORT checklist consists of 25 main items with several sub-items. Most items were modifications of items contained in the CONSORT 2010 checklist or the CONSORT extension for crossover trials, but 1 sub-item pertaining to euthanasia was created *de novo*.

**Conclusion:**

The methods and processes used to develop this guideline represent a novel departure from those used to create other reporting guidelines, by using a virtual format. The use of the PetSORT statement should improve reporting of trials conducted in client- and shelter-owned dogs and cats and published in the veterinary research literature.

## 1. Introduction

Veterinarians, regulators, and other veterinary health professionals increasingly are expected to make evidence-based decisions, where the evidence comes from research (1). When evaluating the efficacy of interventions where it is ethical and feasible to allocate study subjects to intervention groups, randomized controlled trials (RCTs) provide the highest level of evidence of the primary research designs (2, 3). However, evaluating the potential for bias and interpreting the results of an RCT requires comprehensive reporting of the trial design and conduct. Earlier studies in human healthcare illustrated inadequacies in reporting of RCTs (4–9). This led to the creation of the Consolidated Standards of Reporting Trials (CONSORT) statement for reporting of two-group parallel design RCTs, first published in 1996 (10), revised in 2001 (11) and again in 2010 (12). The CONSORT statement was developed by expert consensus and consists of a checklist of 25 items that should be reported in all RCT reports. An accompanying elaboration document provided details on the rationale for including each item, as well as examples from the literature illustrating comprehensive reporting for the items (13). Meta-research studies have shown that reporting of RCTs in human healthcare has improved following the publication of the CONSORT statement (14, 15).

In the years since the CONSORT statement was first published, a number of extensions have been published to address variations in trial design or different types of interventions. These include CONSORT extensions for crossover trials (16), multi-arm parallel group randomized trials (17), non-inferiority and equivalence trials (18), and reporting of RCT abstracts in journals or conference proceedings (19).

Formal reporting guidelines developed by expert consensus have been published for trials conducted in animal publications. The REFLECT statement (20) provides guidelines for reporting clinical trials conducted in livestock populations. In addition to providing guidance for reporting some features of livestock trials that differ from human trials, the elaboration document (21) also provides relevant examples from the livestock trial literature. There is evidence that reporting of swine intervention trials (22) and bovine respiratory disease trials (23) has improved since the publication of the REFLECT statement.

The ARRIVE statement for reporting of *in vivo* experiments in animals originally was published in 2010 (24), with a revised ARRIVE 2.0 published in 2020 (25, 26). An updated document with explanations and examples of the items in ARRIVE 2.0 also was published in 2020 (27). The focus of ARRIVE 2.0 is on comparative studies; thus, studies using dogs and cats would be within the scope of these guidelines. However, trials including dogs were only used in two of the examples in the explanation document, and in both instances the experiments used dogs as animal models of human illness (27). None of the examples described experiments conducted in cats.

Early evaluations of the quality of reporting of clinical trials in dogs and cats have found substantive deficiencies in the reporting of small animal trials (28, 29). In the 2010 evaluation, in addition to documenting inadequate reporting, the authors reported an association between inadequate reporting and trial results; an increased proportion of positive treatment effects within a trial was associated with not reporting key features such as the method used to generate the random allocation sequence, the use of double blinding, and the eligibility criteria for animals. In an updated evaluation of trials published after 2015 in populations of dogs or cats, ~1/5 of published trials used a crossover design (257 / 1190) (30). When evaluating the quality of reporting in 200 trials published during 2019, the authors noted that some trial features, such as method of allocation to intervention group, were well reported. However, despite the availability of relevant reporting guidelines such as CONSORT and ARRIVE, there still were substantive deficiencies in the reporting of trials in dogs and cats (30). The reason for continued inadequacies in reporting is unknown. However, it is possible that individuals conducting trials in dogs and cats are not aware of the existence of relevant guidelines such as CONSORT, the CONSORT extension for cross-over trials, ARRIVE, or REFLECT, or that the explanations and examples in those guidelines are not sufficiently relevant to the trial conditions that they experience.

Thus, there is a need for reporting guidelines for owned dogs and cats, both to modify reporting items to address nuances between trials in livestock, humans, or for biomedical purposes and to provide relevant examples of good reporting for researchers conducting trials in owned dogs and cats. Given the prevalence of cross over designs, there may be value in combining the reporting of parallel and crossover trials into a single guideline to facilitate access for researchers.

Therefore, the objective of this work is to describe the methods used to develop reporting guidelines for parallel group and crossover trials conducted in client- and shelter-owned dog and cat populations (PetSORT). A separate companion paper, the PetSORT explanation and elaboration document (31), provides the methodologic background for the items contained in the PetSORT statement as well as illustrative examples of appropriate reporting. We strongly recommend that the PetSORT checklist be used in conjunction with the explanation and elaboration document for reporting of all trials conducted in dog and cat populations.

## 2. Methods

The process used for developing reporting guideline statements have been documented and published previously (32–34). Typically, this process has included an in-person consensus meeting occurring over a period of several consecutive days during which members of the working group discuss and reach agreement on items to be included in the guidelines. For this project, however, travel restrictions and lockdowns associated with the COVID-19 pandemic precluded using this approach.

### 2.1. Steering committee

A steering committee of four members (co-authors AR, JS, LS, and AO'C) was formed with the express purpose of developing a reporting guideline for trials that involve client- and shelter-owned dog and cat populations. Two of the steering committee members had previously led initiatives to develop reporting guidelines in veterinary medicine (JS, AO'C). The steering group first met in June 2020, and ultimately were responsible for development of the initial checklist, which was based on the items included in the 2010 CONSORT statement (12), as well as items from the CONSORT extension for crossover trials (16). The steering committee intentionally combined items pertinent to both parallel and crossover trials into one consolidated checklist in order to improve ease of use for investigators. This committee then identified and invited potential participants, coordinated the collection of participants' opinions on each item included in the guidelines, revised and recirculated modifications to participants, and were responsible for all subsequent steps involved in preparation, revisions, and publication of the manuscripts associated with this work.

### 2.2. Identification of experts

The aim of the steering committee was to include experts with experience in a wide variety of areas in the consensus group, but all having familiarity with design, analysis, and publication of trials. For this guideline, trials were defined as a controlled experiment where there were at least two groups, the investigator controlled allocation to intervention groups, and disease or outcome occurrence was natural rather than induced and conducted in client- and shelter-owned dogs and cats. Diversity was sought in terms of specific areas of content expertise (e.g., veterinary specialists were included from the specialty areas of oncology, nutrition, internal medicine, ophthalmology, emergency medicine, pharmacology, surgery, and public health) as well as areas of employment (e.g., academia, regulatory agencies, public and private research companies, and clinical practices). Representation from multiple countries was intentionally prioritized and an effort was made to include participants with relevant editorial experience.

Previously published work that included the use of a consensus group reported imposing size limitations on the total number of experts to include in the panel based upon funding and the need to allow for active participation in conversation (34). Due to the virtual nature of this project, the steering committee decided not to cap the total number of participants included in the expert panel. Instead, an initial group of 32 individuals with qualifying expertise were identified by the steering committee and invited to participate in this work *via* email. The initial group was selected based upon several criteria including their area of expertise, history of publication of controlled trials, previous participation in reporting guidelines consensus groups, geographic location, and the perceived impact reporting guidelines would have on their work. Only two of the experts initially invited had previously published with any of the steering committee members. All invitees, whether they agreed to participate or not, were asked to nominate other experts to participate in this work in order to ensure participation from a robust consensus group.

### 2.3. Consensus process

The email invitation sent to experts requested that individuals who wished to participate complete a modified Delphi survey indicating their thoughts on the initial PetSORT checklist items anonymously. For each item participants were first asked whether they agreed the item should be included. If the participant thought the item should be included, they were further queried if the item was worded appropriately or if they had suggestions about how to modify the item. Experts were also invited to offer additional comments on each item and could include any feedback they thought necessary to communicate to the steering committee. Surveys results were collected using Qualtrics, a web-based survey platform.

The steering committee decided that consensus would be reached when 87.5% of experts agreed upon the exclusion or inclusion of an item as it was currently worded i.e., at least 49 of the 56 experts agreed. Items that the experts agreed should be included, but did not reach consensus in terms of the wording of the item, were modified by the steering committee to address concerns and comments from the expert panel and recirculated for another round of voting that included the contextual reasons and comments provided by experts in the previous survey responses. This was repeated until consensus was reached about the wording for each item. The identities of the experts involved in this process were not revealed until after consensus was reached.

### 2.4. Preparation of reporting guidelines

The steering committee compiled the proposed modifications to the initial checklist developed by the steering committee and collated the comments and suggested revisions and used these to develop the final reporting guidelines for use in reporting trials conducted in dog and cat populations. A draft of the explanation and elaboration document was then prepared by the steering committee and circulated among all participants for input. Feedback from all participants was incorporated into the final version of the manuscript by the members of the steering committee.

## 3. Results

**Figure 1:**
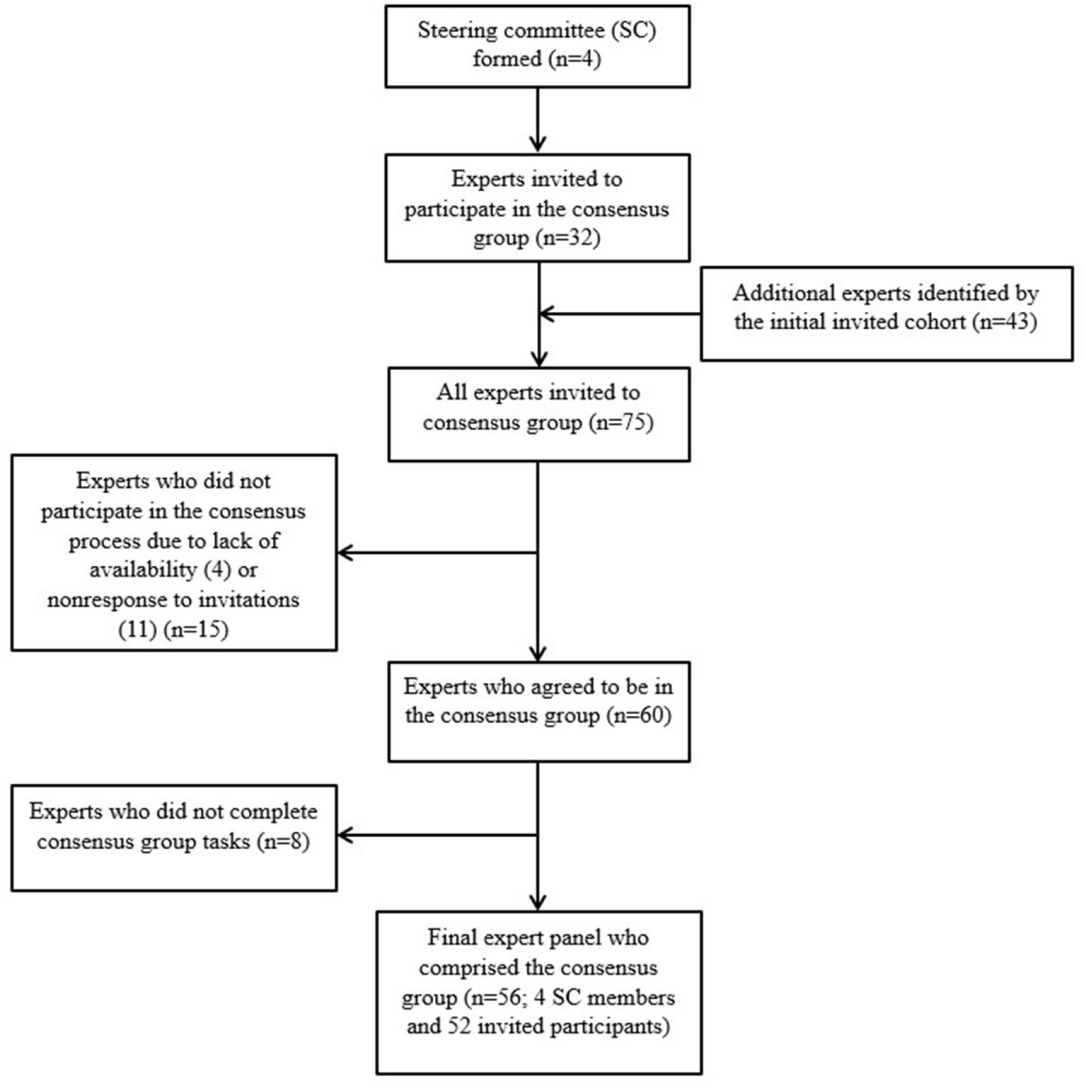
Consensus group participant flow chart during creation of the expert panel.

Seventy-five experts were invited to participate in the consensus group and 52 accepted the invitation and completed all tasks (Figure 1). All four of the steering committee members participated for a total of 56 members in the consensus group. The methodological expertise of the participants included trial design, epidemiology, statistics, regulatory medicine, clinical practice, systematic review and meta-analysis. The majority of experts (*n* = 43, 76.8%) were employed in the United States, 10 (17.9%) were employed in the United Kingdom, and 3 (5.4%) were employed in Canada, Germany, and Australia. Academicians accounted for the majority of the consensus group (*n* = 46, 82.1%), 6 (10.7%) members of the group worked for a government agency, and 4 (7.1%) worked in industry or private practice.

The steering committee proposed an initial set of guidelines that included 25 main items which resulted in 38 individual items when sub-items are counted. There was consensus among the experts to include all 38 items in the final checklist for PetSORT but only 18 of the initially proposed items were accepted by the consensus group as they were worded by the steering committee. These 18 items were not further modified by the expert group. Consensus about the wording proposed by the steering committee was not reached for 20 of the items initially presented to the consensus group and revisions and modifications were made in an iterative process to reflect the consensus of the group.

Compared to the original CONSORT 2010 guidelines for parallel trials, only 2 of the items (2b and 7b) included in PetSORT required no modifications by the steering committee (Table 1). Eighteen of the items included in PetSORT (1a, 2a, 6a, 6b, 7a, 8a, 8b, 10, 11b, 12a, 14a, 14b, 15, 17a, 17b, 18, 20, and 21) had only minor modifications made from the CONSORT 2010 statement for parallel trials in order to clarify or add more details specific to the veterinary community. Seventeen PetSORT items include more substantial changes from the CONSORT 2010 statement for parallel trials (Table 2). One item, 6c, was developed specifically to address the need for inclusion of discussion in reporting of trials involving dogs and cats regarding consultation about and performance of euthanasia and the possible impact of losses due to euthanasia on the outcome of trials conducted in pets.

**Table 1:** PetSORT checklist of information to include when reporting a randomized trial.

**Section/Topic**	**Item no**.	**Checklist item**	**Reported on page no**.
**Title and abstract**			
	1a	Identify the study as a randomized trial in the title.	
	1b	Summarize the objective, trial design, primary outcome(s), study population, intervention, results, and conclusions/clinical relevance.	
**Introduction**			
Background and Objectives	2a	Give scientific background and explanation of rationale.	
	2b	Specify objectives or hypotheses.	
**Methods**			
Trial design	3a	Describe trial design (such as parallel, factorial, crossover) and the level of allocation of the intervention (such as animal, litter, kennel). For crossover trials, description of the number and duration of intervention and washout periods.	
	3b	Report any changes to methods after trial commencement (such as eligibility criteria), with reasons.	
Participants	4a	Report eligibility criteria for animals and their caregivers (includes owners of pets and custodians of shelter animals) at all organizational levels (such as animal or veterinary clinic). State whether animals were shelter-owned or client-owned.	
	4b	Describe settings and locations where the data were collected. Describe sources of clustering (such as multiple veterinary practices or group housing).	
Interventions	5	Describe interventions for each group with sufficient details to allow replication. Describe the unit of allocation (such as body part (eye), individual animal, litter).	
Outcomes	6a	Completely define pre-specified primary and secondary outcome measures, including how, when, and by whom they were assessed.	
	6b	Describe any changes to trial outcomes after the trial commenced, with reasons.	
	6c	If the outcome of interest (such as survival time) could be differentially impacted by euthanasia, describe methods used to reduce bias in study results (such as standardized criteria or counseling for euthanasia).	
Sample size	7a	Provide a sample size calculation or a justification for the sample size if a calculation was not performed.	
	7b	When applicable, explain any interim analyses and stopping guidelines.	
**Randomization:**			
Sequence generation	8a	Describe the method used to generate the random allocation sequence.	
	8b	Describe the type of randomization and include details of any restriction (such as stratification, blocking, and block size) used.	
Allocation concealment	9	Describe the steps taken to conceal the allocation sequence until interventions were assigned.	
Implementation	10	Describe who generated the random allocation sequence, who enrolled study subjects, and who assigned them to interventions.	
Blinding or masking	11a	Report which individuals (such as caregivers, investigators, outcome assessors, data analysts) were blinded/masked after allocation. Provide justification if not blinded/masked.	
	11b	If relevant, describe the similarity of interventions.	
Statistical methods	12a	Describe the statistical methods used to compare groups for primary and secondary outcomes.	
	12b	Describe the methods used for ancillary analyses, such as subgroup analyses and adjusted analyses; report if these were pre-specified in the protocol or unplanned.	
**Results**			
Study subject flow	13a	For each group, state the number of study units (body part, individual animal, or litter) that were assessed for eligibility, randomly assigned, received the intended intervention, and were analyzed for each primary and secondary outcome.	
	13b	Quantify and explain any losses and exclusions after randomization for each group (such as the number per group removed due to adverse events) and for each intervention period in a crossover trial.	
Recruitment	14a	Report the dates defining the periods of recruitment and follow-up.	
	14b	If the trial was discontinued early, provide the reason.	
Baseline data	15	Provide a detailed description (such as a table) of baseline demographic and clinical characteristics that could impact the outcomes for each group.	
Numbers analyzed	16	Report the number analyzed for the primary and all secondary outcomes and whether the analysis was by original assigned groups (intention-to-treat) or per-protocol. Explicitly report the numbers of units lost to follow-up and, if relevant, the number of animals with changed intervention assignments (if relevant for per-protocol).	
Outcomes and estimation	17a	For each primary and secondary outcome, report the results for each group, and the estimated effect size and its precision (such as 95% confidence interval).	
	17b	For binary outcomes, present both absolute and relative effect sizes.	
Ancillary analyses	18	Present the results of any other analyses performed, including subgroup analyses and adjusted analyses, distinguishing pre-specified from unplanned or exploratory analyses.	
Harms	19	Describe the methods for detection of adverse events and report all adverse events (expected, unexpected, and suspected) or unintended effects observed in each group or their absence.	
**Discussion**			
Interpretation	20	Ensure that interpretation is consistent with results, balancing benefits and harms, and considering other relevant evidence.	
Generalizability	21	Discuss generalizability (external validity, applicability) of the trial findings.	
Limitations	22	Discuss trial limitations, addressing sources of potential bias, imprecision, and, if relevant, multiplicity of analyses. Consider potential carryover effects if a crossover trial.	
**Other information**			
Registration	23	State whether the trial was registered and, if so, provide a registration number and name of trial registry. If not, provide a reason for not registering the trial in advance.	
Protocol	24	State if the full trial protocol was finalized a priori and where it can be accessed. Describe any protocol deviations with justification.	
Funding and transparency	25	State sources of funding and other support (such as supply of drugs), role of funders, conflict of interest, ethical approval for human (if applicable) and animal subject use, and quality standards used.	

**Table 2:** PetSORT items paired with the CONSORT 2010 statement for parallel trials item from which the content was substantial changed.

**Item**	**CONSORT 2010 guideline**	**PetSORT**
1b	Structured summary of trial design, methods, results, and conclusions (for specific guidance see CONSORT for abstracts)	Summarize the objective, trial design, primary outcome(s), study population, intervention, results, and conclusions/clinical relevance.
3a	Description of trial design (such as parallel, factorial) including allocation ratio	Describe trial design (such as parallel, factorial, crossover) and the level of allocation of the intervention (such as animal, litter, kennel). For crossover trials, description of the number and duration of intervention and washout periods.
3b	Important changes to methods after trial commencement (such as eligibility criteria), with reasons	Report any changes to methods after trial commencement (such as eligibility criteria), with reasons.
4a	Eligibility criteria for participants	Report eligibility criteria for animals and their caregivers (includes owners of pets and custodians of shelter animals) at all organizational levels (such as animal or veterinary clinic). State whether animals were shelter-owned or client-owned.
4b	Settings and locations where the data were collected	Describe settings and locations where the data were collected. Describe sources of clustering (such as multiple veterinary practices or group housing).
5	The interventions for each group with sufficient details to allow replication, including how and when they were actually administered	Describe interventions for each group with sufficient details to allow replication. Describe the unit of allocation (such as body part (eye), individual animal, litter).
9	Mechanism used to implement the random allocation sequence (such as sequentially numbered containers), describing any steps taken to conceal the sequence until interventions were assigned	Describe the steps taken to conceal the allocation sequence until interventions were assigned.
11a	If done, who was blinded after assignment to interventions (for example, participants, care providers, those assessing outcomes) and how	Report which individuals (such as caregivers, investigators, outcome assessors, data analysts) were blinded/masked after allocation. Provide justification if not blinded/masked.
12b	Methods for additional analyses, such as subgroup analyses and adjusted analyses	Describe the methods used for ancillary analyses, such as subgroup analyses and adjusted analyses; report if these were pre-specified in the protocol or unplanned.
13a	For each group, the numbers of participants who were randomly assigned, received intended treatment, and were analyzed for the primary outcome	For each group, state the number of study units (body part, individual animal, or litter) that were assessed for eligibility, randomly assigned, received the intended intervention, and were analyzed for each primary and secondary outcome.
13b	For each group, losses and exclusions after randomization, together with reasons	Quantify and explain any losses and exclusions after randomization for each group (such as the number per group removed due to adverse events) and for each intervention period in a crossover trial.
16	For each group, number of participants (denominator) included in each analysis and whether the analysis was by original assigned groups	Report the number analyzed for the primary and all secondary outcomes and whether the analysis was by original assigned groups (intention-to-treat) or per-protocol. Explicitly report the numbers of units lost to follow-up and, if relevant, the number of animals with changed intervention assignments (if relevant for per-protocol).
19	All important harms or unintended effects in each group (for specific guidance see CONSORT for harms)	Describe the methods for detection of adverse events and report all adverse events (expected, unexpected, and suspected) or unintended effects observed in each group or their absence.
20/22	Trial limitations, addressing sources of potential bias, imprecision, and, if relevant, multiplicity of analyses	Discuss trial limitations, addressing sources of potential bias, imprecision, and, if relevant, multiplicity of analyses. Consider potential carryover effects if a crossover trial.
23	Registration number and name of trial registry	State whether the trial was registered and, if so, provide a registration number and name of trial registry. If not, provide a reason for not registering the trial in advance.
24	Where the full trial protocol can be accessed, if available	State if the full trial protocol was finalized a priori and where it can be accessed. Describe any protocol deviations with justification.
25	Sources of funding and other support (such as supply of drugs), role of funders	State sources of funding and other support (such as supply of drugs), role of funders, conflict of interest, ethical approval for human (if applicable) and animal subject use, and quality standards used.

## 4. Discussion

This work describes the development of reporting guidelines for use when reporting on trials conducted in client- and shelter-owned cat and dog populations. This work was based upon both the CONSORT 2010 statement for reporting parallel group randomized trials (12) and the extension to randomized crossover trials (16). The guidelines represent the consensus of a large group of individuals considered to be experts in trials conducted in dog and cat populations. The results of this work, therefore, represent consensus of expert opinion.

In concordance with the CONSORT statement, the intention of these guidelines is to provide guidance for authors when describing the design and results of trials. However, these guidelines are also useful for editors and peer reviewers assessing the comprehensiveness of reporting when considering suitability of trials for publication, researchers conducting systematic reviews, and readers attempting to assess internal and external validity of the trials being reported.

Like the CONSORT statement, the PetSORT guidelines are not intended to be prescriptive regarding the order of reporting. The items were generally ordered to correspond to the CONSORT statement, which follows the typical order of sections within a scientific manuscript. Thus, while it is important that all of the relevant items on the checklist are addressed in sufficient detail within a manuscript, that content does not necessarily need to correspond to the section in which the item number is located on the checklist. It is also important to note that the PetSORT statement is not intended to be used as a tool to assess the quality of the research design or execution of the trial.

It is of note that the majority of the participants in the consensus group were employed in the United States and this may be considered a selection bias. Similarly, most of the consensus group was employed in an academic setting. The effect of the bias, if present, cannot be determined due to the survey responses having been collected anonymously. It might also be considered a limitation that people contacted to participate in this work were asked to nominate other experts to the group. This may have resulted in invitations being extended based upon personal or professional connections which could have resulted in inclusion of experts who think similarly about these concepts. However, conducting this work virtually rather than face-to-face allowed for a larger and potentially more diverse group of individuals to participate in the consensus process and we feel the effect of personal connections with the original group of invitees was mitigated due to the large size of the expert panel.

It was agreed a priori that the exact number of experts in agreement on each item would not be published which is in keeping with standards of guideline development (35–37). The steering committee felt that publication of the specific figures would detract from the purpose of publication of a consensus statement, which is to define the general agreement of the group. However, anonymized aggregated data including individual responses and approval rates can be requested from the corresponding author.

When used with the PetSORT explanation and elaboration document, we expect these guidelines will lead to improved reporting of trials conducted in client- and shelter-owned dog and cat populations.

## Data availability statement

The original contributions presented in the study are included in the article/supplementary material, further inquiries can be directed to the corresponding authors.

## Author contributions

AR, JS, LS, and AO'C contributed to the conception and design of this work. AR was primarily responsible for communication between the steering committee and the members of the consensus group. AR and JS drafted the manuscript. LS and AO'C revised the document critically. All authors contributed to the article and approved the submitted version.
